# Multi-class machine classification of suicide-related communication on Twitter

**DOI:** 10.1016/j.osnem.2017.08.001

**Published:** 2017-08

**Authors:** Pete Burnap, Gualtiero Colombo, Rosie Amery, Andrei Hodorog, Jonathan Scourfield

**Affiliations:** a School of Computer Science & Informatics, Cardiff University, UK; b Office for National Statistics Newport, UK; c School of Social Sciences, Cardiff University, UK

**Keywords:** Artificial intelligence, Text analysis, Web-based interaction, Human safety, Suicidal ideation

## Abstract

The World Wide Web, and online social networks in particular, have increased connectivity between people such that information can spread to millions of people in a matter of minutes. This form of online collective contagion has provided many benefits to society, such as providing reassurance and emergency management in the immediate aftermath of natural disasters. However, it also poses a potential risk to vulnerable Web users who receive this information and could subsequently come to harm. One example of this would be the spread of suicidal ideation in online social networks, about which concerns have been raised. In this paper we report the results of a number of machine classifiers built with the aim of classifying text relating to suicide on Twitter. The classifier distinguishes between the more worrying content, such as suicidal ideation, and other suicide-related topics such as reporting of a suicide, memorial, campaigning and support. It also aims to identify flippant references to suicide. We built a set of baseline classifiers using lexical, structural, emotive and psychological features extracted from Twitter posts. We then improved on the baseline classifiers by building an ensemble classifier using the Rotation Forest algorithm and a Maximum Probability voting classification decision method, based on the outcome of base classifiers. This achieved an F-measure of 0.728 overall (for 7 classes, including suicidal ideation) and 0.69 for the suicidal ideation class. We summarise the results by reflecting on the most significant predictive principle components of the suicidal ideation class to provide insight into the language used on Twitter to express suicidal ideation. Finally, we perform a 12-month case study of suicide-related posts where we further evaluate the classification approach - showing a sustained classification performance and providing anonymous insights into the trends and demographic profile of Twitter users posting content of this type.

## 1 Introduction

It is recognised that media reporting about suicide cases has been associated with suicidal behaviour [1] and concerns have been raised about how media communication may have an influence on suicidal ideation and cause a contagion effect between vulnerable subjects [2]. With the advent of open and massively popular social networking and microblogging Web sites, such as Facebook, Tumblr and Twitter (frequently referred to as social media), attention has focused on how these new modes of communication may become a new, highly interconnected forum for collective communication and, like news media reporting, lead to contagion of suicidal ideation or at least have the effect of normalizing the desire to self-harm [3].

The concerns about suicide-related communication in social media assume that statements of suicidality within social media platforms are indicators of actual suicidal distress in vulnerable individuals who are posting this material, therefore the affective quality of suicide talk in social media needs to be identified and perhaps responded to. There is some limited evidence of an association between online exposure to suicide-related material and offline suicidal ideation [4] although research on this issue is underdeveloped and online prevention is in its infancy.

Social science and medical research have investigated the impact that communication on the topic of suicide via the World Wide Web may have on vulnerable subjects, with particular attention to the younger generation. [5] conducted a qualitative study by interviewing young adults who engage in suicidal behaviours and use websites dedicated to these themes. [6], [7] also conducted online searches for Web resources containing suicide-related terms and describing suicide methods. They presented a qualitative analysis of the resources they discovered and concluded that, although neutral and anti-suicide Web sites occurred most frequently, pro-suicide forums and Web sites encouraging suicidal behaviour were also present and available, suggesting that more prevention plans specifically focused on Web resources are required. Building on this, [8] have reviewed online suicide intervention and prevention literature, concluding that there is a lack of published evidence about online prevention strategies and more attention is required to develop and evaluate online preventative approaches. [9] also studied the impact of Facebook suicide notes on suicidal behaviour, reporting that it was not yet clear to what extent suicide notes on online social media actually induce copycat suicides. They note that suicide and social media effects deserve further evaluation and research.

Other studies have focused on the written communication of suicide on the Web via bulletin boards [10], newsgroups [11], chat rooms [12], and web forums [13]. These are mostly qualitative analyses and where quantitative data are used in web-related suicide studies, they tend to rely solely on human classification, which is difficult to implement at scale. Computational methods have only been used in a small number of suicide communication studies.

Some studies report a positive correlation between suicide rates and the volume of social media posts that may be related to suicidal ideation and intent [14], [15]. There is also a developing body of literature on the topic of identifying suicidal language on Twitter [16], [17], but very few attempts to use machine classification to automatically identify suicidal language and differentiate between this and other forms of suicide-related communication, such as awareness raising and reporting of suicides. The differentiation is a requirement for the purposes of analysing the characteristics of suicidal ideation on social media. [18], [19] study depression and other emotional states expressed via social media. Suicidal language is likely to include emotive content and possible signs of depression but we do not suggest depression and suicidal ideation are synonymous in this paper. Two recent papers presented the results of Twitter studies aiming to classify ‘risky’ language [20] and levels of ‘distress’ [21] – both reporting classification performance that has potential for improvement (around 60%–64%). An important step in providing support to suicidal social media users is to understand how suicidal ideation is communicated. Recent studies have shown that people are more likely to seek support from non-professional resources such as social media, rather than risk social stigmatisation by seeking formal treatment [21].

Thus, our study aims to contribute to the literature on understanding communications on the topic of suicide in social media by (i) creating a new human-annotated dataset to help identify features of suicidal ideation, (ii) creating a set of benchmark experimental results for machine learning approaches to the classification of suicidal ideation, and (iii) developing a machine classifier capable of distinguishing between worrying language such as suicidal ideation, and flippant references to suicide, awareness raising about suicide and reports of suicide. This last contribution is especially relevant to quantify actual volumes of worrying language on social media for the purposes of understanding risk to human safety, as opposed to all references to suicide. The research presented in this paper comprises an analysis of data collected from the microblogging website Twitter, the text of which has been classified into one of seven suicide-related categories by a crowdsourced team of human annotators. We then use a range of machine learning classification methods to identify suicidal ideation in tweets and analyse the predictive features of suicidal ideation to help explain the language used by perceived suicidal social media users. We apply this to a data set collected from Twitter over 12 months, to further test the most effective classifier, observe trends over time and estimate demographics.

## 2 Related work

The Durkheim project is aiming to mine social media data to identify markers of harmful behaviour [22]. The project will study a group of US war veterans who will opt-in to share their Twitter, Facebook and LinkedIn posts over time. There are so far no publicly available results from this study but the group has recently published the results of a suicide prediction task, using text from the clinical notes of US war veterans to identify text-based statistically significant *signals* of suicidality, with around 60% accuracy [23]. They found clinical notes of people who had died through suicide frequently recorded behaviours indicative of fear, agitation and delusion.

Written text has also been analysed in a number of recent studies that have analysed clinical conversations [24] and suicide notes to develop machine classifiers to identify topics and emotions expressed by people who have taken their lives [25], [26], [27], [28], [29], [30]. Many of these papers attempt to classify text at a sentence level, which would suggest short strings much like those that would be posted to social media. However, suicide notes are written by people who have accepted suicide and then go on to harm themselves, whereas the current research is particularly interested in identifying suicidal thinking or ideation prior to self-harm, which may differ from the language used in suicide notes. Additionally, handwritten notes, even at sentence level, are not constrained by string length. Twitter posts are limited to 140 characters, which forces authors to use short, informal language that may differ from the way they would normally express feelings on paper. Finally, social media data are noisy, contain a broad range of topics, and language use varies over time. These features arguably make the task of classifying suicidal ideation more complex than it would be in a discrete recording of pre-suicide thoughts and feelings in a suicide note.

A small number of studies have investigated the communication of suicidal ideation on social media. However, they are mainly focused on a comparison with national death rates. For example, in Korea [14] and the US [15] research has attempted to identify a positive correlation between the frequency of suicide-related posts on social media and the number of recorded suicide cases. Suicide related posts were identified using a set of keywords relating to general concepts such as suicide and depression [14] or relating to specific risk factors [15]. [31], [32], [33] consider online platforms such as *r*/*SuicideWatch* on *Reddit* and applies topic analysis and linguistic features to identify behavioural shifts between mental health issues and suicidal ideation, thus highlighting the risks of supposedly helpful messages in such support online forums. [34] investigated the characteristics of the authors of Tweets containing suicidal intent or thinking, through the analysis of their online social network relationships and interactions rather than focussing on the text in the posts.

These behavioural changes can be also triggered by external factors as celebrities deaths [35], [36], thus backing the results in [14]. Similarly, [37] found statistical correlations between suicide rates in the Japanese population and high peaks of social media posts related to celebrities suicides. [38] also focuses on social media reactions to high profile deaths by suicide but uses a semi-automated procedure that replaces manual coding with a combination of crowdsourcing and machine learning.

[16] analysed the Twitter posts of a person who had recently died through suicide. They studied the posts sent in the twenty-four hours prior to death, finding an increase in positive emotions (though not statistically significant) and a change in focus from the self to others as the time of death approached. As this was only a single person study, and given the fact the person had attempted to make the posts rhyme (thereby perhaps using different language to achieve this), the authors propose larger studies of a wider range of Twitter posts. They used the Linguistic Inquiry and Word Count (LIWC) software to identify parts of speech, emotional words and cognitive processes among other concepts [39]. LIWC was also used in [21] as a sampling technique to identify ’sad’ Twitter posts that were subsequently classified using a machine learning into levels of distress on an ordinal scale, with around 64% accuracy in the best-case.

Also studying linguistic features of suicidal ideation, [17] used an online panel of young (early 20 s) Twitter users to examine the association between suicide-related tweets and suicidal behaviour. They identified that particular phrases such as ‘want to commit suicide’ were strongly associated with lifetime suicide attempts, the most powerful predictor of future suicide. They also noted that other phrases that suggest suicidal intent, such as ‘want to die’, were less strongly associated. The variation here could suggest the flippant use of such phrases on social media when having a bad day – hence the additional challenges posed to classification of suicidal ideation on social media. In another example, [20] used machine learning to classify ‘risky’ and ‘non risky’ Tweets, as defined by human annotators, with an accuracy of around 60%. They created word lists to represent a number of topics and emotions related to suicide, finding references to insults, hurt and bullying in the ‘risky’ category. [40] also considers on-line social media but adopts a different approach than linguistic to identify possible suicidal content through friendship ties to users that are known to be active on user-defined community related to suicide.

Finally, [41] used human coders to categorise Twitter posts as ‘strongly concerning’, ‘possibly concerning’ and ‘safe to ignore’. The researchers subsequently used machine learning to develop a classifier for the ’strongly concerning’ posts, 80%   of which were correctly classified. The most effective algorithm out of those trialled was Support Vector Machines (SVMs) with Term Frequency weighted by Inverse Document Frequency (TFIDF), not filtered for words with little information. This study focuses on a binary classification of strongly concerning vs.not and does not take into account the other forms of suicide-related communication in social media. Similarly, [42] classify text from Twitter users as suicidal or non-suicidal using affective markers and machine classification algorithms – stopping short of examining texts for other forms of suicidal communication.

## 3 Data

### 3.1 Data collection and annotation

Rather than manually developing a word list to represent suicidal language, we generated a lexicon of terms by collecting anonymised data from known suicide Web forums, blogs and microblogs, and asking human annotators to identify whether it contained references to suicidal ideation. First we collected user posts from four known Web sites identified by experts in the field [6], [7] as being used to discuss suicidal themes for support and prevention. The selected Web sites either had dedicated sections,^1^^,^^2^ or are specifically designed for suicidal discussions.^3^^,^^4^ Then we collected data from microblogging site Tumblr^5^ – specifically, content containing self-classified suicidal ideation (i.e. text posts ‘tagged’ with the word ‘suicide’).

For each of the resulting Web sites we then collected an equal number of 200 posts, retrieved in chronological order, with a total of 800 text posts. These posts, and 1000 posts randomly selected from the Tubmlr sample, were subsequently human annotated using the crowdsourcing online service Crowdflower^6^. To avoid difficulties in the annotation of long pieces of text we discarded posts having a length greater than the five percent longer than the average post length for each of the websites considered. Human annotators were asked to identify content containing suicidal ideation using a binary criteria by answering the question ‘Is this person suicidal?’.

We then applied the Term Frequency/Inverse Document frequency (TF.IDF) method to the corpus of annotated documents in order to identify terms that appear frequently in the suicidal ideation class but appear with less frequency in the non-ideation class. This process identifies terms that can be used to distinguish between the two classes. In the TF.IDF process we considered n-grams of 1 to 5 words in length, and ranked the top 500 terms. These terms were further analysed by two experienced suicide researchers to remove terms not specifically related to suicide, as well as duplicate keywords. This resulted in a final list of 62 keywords and phrases that suggested possible suicide intent. Illustrative examples are *asleep and never wake, don’t want to exist* and *kill myself*. These search terms were then used to collect data from Twitter via the Twitter Streaming Application Programming Interface (API).

Twitter data were collected for a continuous period of six weeks from 1st February 2014 using the suicide-related search terms, resulting in a dataset of over four million posts. In parallel we monitored traditional media over the same period to identify the names of reported suicide cases in England. We then retrieved a second data set from Twitter using the name and surname of the deceased as search keywords. Here, the underlying idea was to collect different types of posts with a connection to suicide other than those more directly expressing suicidal ideation (which was the aim of the first dataset collection). All names were removed from the text before analysis.

Following the data collection we produced a random sample of 1000 tweets from both datasets, with 80% of posts from the collection of suicide related search terms, and the remaining from the ‘names’ dataset. The human annotation task was repeated using the same crowdsourcing service. This time human annotators were asked to classify data into either one or more of the six suicide related categories listed below, or into the seventh category representing tweets that cannot be classified into any of them. This coding frame was developed with expert researchers in suicide studies to capture the best representation of how people generally communicate on the topic of suicide.

Text annotation can be a subjective task, so to limit the amount of subjectivity we required at least 4 human annotations per tweet as per the convention in related research [43]. CrowdFlower provides an agreement score for each annotated unit, which is based on the majority vote of the trusted workers [44]. Because the crowdsourcing service continues to recruit workers until the task is complete, there is no guarantee that all workers will annotate the same set of units. Therefore, we cannot calculate traditional inter-rater reliability (IRR) scores, such as Krippendorf’s Alpha or Cohen’s Kappa to determine agreement between all annotators. However, CrowdFlower has been shown to produce an agreement score that compares well to these classic measures [44]. Based on the output from our annotator task we can determine agreement on each unit. The purpose of the experiments performed in this paper are to establish the accuracy of a machine classifier when assigning tweets to a particular class of suicidal communication, and thus it is the agreement score for the unit of analysis (each tweet), and not the overall human agreement for all units that is important for validation. We removed all tweets with less than 75% agreement - again, following established methods from related research [43], and discarded any where less than three out of four annotators (75%) agreed on the dominant class for each tweet. Annotators were allowed to select multiple class labels and the majority choice was taken. The distribution of tweets to classes from c1 to c7 is shown in Table 1. Note that the dominant class was flippant and improper use of suicide-related phrases and expressions, with actual suicidal intent or thinking being in the minority (about 13% of the total). The fact that four people unknown to each other and without being influenced by each other’s annotations could agree to this level would suggest that it is possible for human annotators to agree on what constitutes the language of suicidal ideation, and what is simply a flippant reference to suicide. The resulting dataset of 816 Tweets was subsequently used to train a machine learning classifier (details are provided in the next section), which is only slightly below the dataset sizes of other similar analyses of emotive content on social media e.g. [43], [45], [46], [47].

**Table 1:** Types of suicidal communication with relative % proportion in dataset.

Class	Description	% of dataset
c1	Evidence of possible suicidal intent	13
c2	Campaigning (i.e. petitions etc.)	5
c3	Flippant reference to suicide	30
c4	Information or support	6
c5	Memorial or condolence	5
c6	Reporting of suicide (not bombing)	15
c7	None of the above	26

### 3.2 Feature preparation

We used the text of the tweets in order to train and test a number of machine classifiers to identify suicidal ideation and differentiate between this and other types of suicide-related communication, including flippant references to suicide. Three features sets were derived from the text as follows:

• Features representing *lexical characteristics* of the sentences used, such as the Parts of Speech (POS), and other *language structural features*, such as the most frequently used *words* and *phrases*. These are standard features used in most text mining tasks. References to self and others are also captured with POS – these terms have been identified in previous research as being evident within suicidal communication;
• Features representing *sentiment, affective and emotional features and levels* of the terms used within the text. These were incorporated because of the particularly emotive nature of the task. Emotions such as fear, anger and general aggressiveness are particularly prominent in suicidal communication [20]
• Features representing idiosyncratic language expressed in short, informal text such as social media posts within a limited number of characters. These were extracted from the annotated Tumblr posts we collected to try and incorporate the language used on social media that may not be identified using standard text mining features.

#### 3.2.1 Feature set1

For the first set of features, and part of the second set, we derived features used in [26], published within the special issue on *sentiment analysis of suicide notes* [48]. We will refer to this set of features as *Set*1. More specifically, the *Set*1 feature set included the following:

• *Parts of speech*. We used to the Stanford Part-Of-Speech (POS) Tagger^7^ to assign each word in a Tweet a POS label. Examples are nouns (broken down into singular, plural, proper), verbs (specifying tenses such as present, past and present participle), 1st vs 3rd person references, adjective and adverbs (comparative, superlative), pronouns (personal, possessive), as well as other tags representing conjunctions, determiners, cardinal numbers, symbols, and interjections. For each of POS we considered the frequency of each in a Tweet as a feature.
• *other structural features*. For this we considered the inclusion of negations in the sentence (total number), the specific use of a first person pronoun (either singular or plural), and external communication features such as the inclusion of a URL in a tweet or a mention symbol (indicating a retweet or reply).
• *General lexical domains*. These features represent general lexical categories such as home, religion, psychology, sociology, etc. These were extracted using WordNet Domains labels,^8^
• *Affective lexical domains*. These are a set of categories specifically related to domains representing ’affective’ concepts. These include concepts representing moods, situations eliciting emotions, or emotional responses such as joy, anger, grief, sadness, enthusiasm, surprise, love, hate, and happiness; but even more specific sub-categories such as amicability, belligerence, bad-temper, unrest, and trepidation; and opposites such as positive-negative concern, negative fear, positive-negative suspense, self-esteem, self consciousness, self-pity, and self-deprecation. These are very appropriate for the specific language we are investigating in this study.
• *Sentiment score*. Using SentiWordNet^9^ each words is assigned a score between zero and one for both positivity and negativity. The sum all words in a Tweet were used as features.
• *Words*. The most frequently used *words* and *n-grams* in terms of (first 100) unigrams, bigrams and trigrams contained in the training set.
• *Keyword list*. We also included each of the 62 keywords derived from the Web form text that were used for the pre-filtering search (e.g. ‘asleep and never wake’, ‘don’t want to try anymore’, ‘end it all’, ‘isn’t worth living’, ‘my life is pointless’, ‘kill myself’, ‘to live any more”, ‘want to end it’, ‘want to disappear’, ‘want to die’, etc.). Each of the search terms were included as individual features together with one global binary feature representing the inclusion of any of them in a Tweet.

#### 3.2.2 Feature set 2

Given the psychological and emotional expressiveness of suicidal ideation, we then explored a second set of features by using the Linguistic Inquiry and Word Count *LIWC* text analysis software [39] to extract more specific labels representing affective emotions and feelings within the text. We refer to these features as *Set*2. These include a more extensive breakdown of categories that may be more suitable for the particular language of emotional distress that we would expect to be present in suicidal ideation. Examples are related to death, health, money, religion, occupation, and achievement, senses (e.g. feeling, hearing, seeing), and three other groups of terms related to ‘cognitive mechanisms’, ‘affect’, and ‘social words’. These can be further broken down into labels representing family, friends, humans; anxiety, anger, sadness and positive and negative emotions; and terms related to certainty, inhibition, insight, causal, inclusivity and exclusivity. A subset of these features (sadness) were used in [21], but we have incorporated a wider range of the feature set to enable us to distinguish between distress and other forms of suicide-related communication (e.g. grief, support and reporting).

#### 3.2.3 Feature set 3

Next, due to the noisy nature of social media, where short, informal spelling and grammar are often used, we developed a set of regular expression (RegEx) and pattern matching rules from our collection of suicide-related posts collected from social networking website Tumblr. We refer to these features as *Set*3. These were annotated as part of the human annotation process conducted earlier and introduce language from short informal text related to the six suicide related categories to assist the classifier. Examples of these expressions for each class (numbered 1–6 here) include:

*1*: ‘.+((\cutting ∣\depres∣\sui)∣\these∣\bad∣\sad).+ (\thoughts∣ \feel).+’ to represent phrases such as ‘suicidal / cutting / bad / these . . . thoughts / feelings’; *‘.+*\*wan*\*w.+d[ie].+’* for expressions as ‘want/wanted/wanting to die’; *‘.+*\*end.+ (*\*all*∣\*it*∣\*life).+’* for sentences with ‘end/ending it all’ and ‘end my life’; and *‘.+ (can.+*∣*don.+*∣\*take).+(*\*go*∣\*live*∣\*anymo*∣ \*cop*∣\*alive).+’* covering a wide range of phases including ‘can’t take anymore’, ‘can’t/don’t want to live/cope anymore’, ‘don’t want to be alive’, ‘can’t take it anymore’, and ‘can’t go on’. In addition, we added a list of individual words and n-grams including ‘trigger warning’, ‘tw’, ‘eating disorder’, ‘death’, ‘selfharm’ and ‘self harm’, ‘anxiety’, and ‘pain’.

*2: ‘.+(*\*need*∣\*ask*∣\*call*∣\*offer).+*\*help.+’* related to phrases as ‘call/offer for/of help’ and individual terms as ‘shut’ (e.g. website shut down) and ‘stop’ (e.g. bullying).

*3: ‘.+(*\*kill*\*hat*\*throw)’* for phrases including ‘kill/killing /hate myself’, *‘.+(*\f**k.+’ for swearwords such as ‘f**k/ f**king’, *‘.+ (*\*boy*\*girl).+(*\*friend)’* for expressions with ‘boy-friend’ and ‘girlfriend’, and *‘.+(*\ *just)*\*.+(*\*like).+’* covering expression including ‘just’ . . . like’. In addition, some words related to general topics such as ‘work’ and ‘school’ have also been included since they are representing contexts more favourable to flippant language rather than genuine expression of distress and suicidal intent.

*4: ‘.+(*\*talk*∣\*speak).+*\*to.+(*\*one*∣\*some*∣\*any).+’* related to phr-ases as ‘talk / speak to someone/somebody’ and words such as ‘web’, ‘blog’, ‘health’ , and ‘advice’.

*5: ‘.+miss.+(*\*you*∣\*her*∣\*him).+’* related to phrases such as ‘miss/missing you/her/him’ and *‘.+(*\*kill*∣\*die*∣\*comm).+(day| month|year).+’* to represent specific time references.

*6: ‘.+(*\*took*∣\*take).+*\*own.+*\*life.+’* covering expressions including ‘took/taken his/her own life’ and words related to suicide methods such as ‘hanged’, ‘hanging’ and ‘overdose’.

Note that the regular expressions included in the third class representing flippancy were also identified within those related to the first suicidal class (and vice versa). However, we decided to associate RegExs to only one of the two classes according to the nature of the annotated tweets, for example phrases as ‘hate myself’ or ‘kill myself’ were frequently associated with flippant posts whereas terms such as ‘wanted to die’ and ‘want to end it’ were more likely to be included in tweets containing evidence of suicidal thinking.

#### 3.2.4 Data-driven features

We built a fourth feature set that we will refer to as the *combined* set, incorporating the union of all of the features in the three previous groups. Given the large number of features associated with each tweet, and potential for colinearity between features in the *combined* set, we applied *Principal Component Analysis (PCA)* as a dimension reduction procedure to convert the set of all possibly correlated variables within the *combined* set into a new set of linearly uncorrelated features (called principal components).

The text of the tweets was also incorporated as a feature set for all experiments. We transformed each Tweet into a word vector using ngrams of size 1 to 5, and retained between 100 and 2000 words (in increments of 100, 300, 500, 1000, 1500 and 2000). The optimum performance was 1-3grams with 500 words retained, and we only present these results in this paper.

## 4 Machine classification method

### 4.1 Baseline experiments

We first conducted baseline experiments using the Weka machine learning libraries.^10^ We used the four derived features sets with the most popular classifiers from the special issue on classification of suicidal topics in [27]. These were *Support Vector Machine (SVM), Rule Based (we used Decision Trees (DT))*, and *Naive Bayes (NB)*.

Support Vector Machines (SVM) have been shown to work very well with short informal text [45], [49], including promising results when classifying other mental health issues [50]. Feature vectors are plotted in high-dimensional space and hyperplanes (lines that separate the data points) are used to try and find the optimum way to divide the space such that the data points belonging to the different human assigned classes are separated. Multiple hyperplanes can be used and the optimal hyperplane will be the line that maximizes the separation between classes. Rule-based approaches are able to iteratively identify the feature from a set of training data that maximises information gain in a classification exercise – or put another way, it quantifies the significance of how using one feature as a rule to classify a tweet as suicidal ideation, reduces the uncertainty as to which class it belongs to. Performing this step multiple times creates a hierarchical and incremental set of rules that can be used to make classification decisions. We used a J48 decision tree (C4.5) to perform rule-based experiments. Finally, given the prevalence of individual words or short combinations of words that would be associated with suicidal ideation, it is logical to incorporate probabilistic classifiers into the experiments as they make classification decisions based on the likelihood of feature occurrence. Specific terms and phrases prevalent in each class can be identified and learned by the classifier. We implemented a Naive Bayes algorithm as a probabilistic approach.

### 4.2 Ensemble experiments

The individual baseline experiments produced a set of results that achieved a reasonable performance but clearly required refining (see Table 2 and dummyTXdummy- further discussion in Section 5). This could suggest that the sample was not large enough to allow the classifier to learn a suitable set of predictive features. It could also suggest the features themselves were either not adequate to represent the latent meaning that human annotators identified when assigning each tweet to a class, or the features were not being suitably utilised during the learning phase. Both sample size and feature set limitations led us to incorporate an *ensemble* classification approach, which enabled us to combine the base classifiers and different methods of feature sampling during the learning phase. There are two very popular ensemble approaches. One is Boosting [51] (e.g. AdaBoost), which aims to ’boost’ the performance of a classifier by iteratively adding a new classifier to the ensemble where each new classifier is trained on data for which the previous iteration performed poorly. An advantage of this is that, for smaller samples, the more difficult to classify instances can be focussed on to improve classifier performance. However, this approach has also been reported to reduce classifier accuracy by forcing new classifiers to focus on difficult data points at the sacrifice of other data. The second popular method is Bagging [52], which takes a bootstrap sample of data points and trains a classifier on each sample, averaging out the probabilities for each class across all classifiers in the ensemble.

**Table 2:** Machine classification results: All classes.

	Classifier				
Feature		NB	DT	SVM	RF
*Set*1	P	*0.694*	0.635	0.692	0.672
	R	0.681	0.641	*0.689*	0.667
	F	0.681	0.637	*0.682*	0.664
*Set*2	P	0.683	0.620	0.698	*0.703*
	R	0.667	0.622	0.696	*0.702*
	F	0.667	0.620	0.689	*0.696*
*Set*3	P	0.694	0.638	0.690	*0.708*
	R	0.679	0.642	0.686	*0.707*
	F	0.680	0.636	0.680	*0.702*
Combined	P	0.674	0.622	0.695	**0.732**
	R	0.659	0.617	0.689	**0.729**
	F	0.658	0.617	0.690	**0.728**
PCA	P	0.607	0.552	0.594	*0.647*
(combined)	R	0.561	0.547	0.586	*0.591*
	F	0.563	0.549	0.581	*0.591*

In [53] the authors propose an ensemble approach known as Rotation Forest (RF), which splits the feature set into a number of smaller sets before sampling from each set and running Principal Component Analysis (PCA) on each subset, creating a number of different principal components for each subset of features, and subsequently building a number of classifiers using these. This approach showed a performance improvement over Bagging and Boosting and provided a logical choice of method to refine our baseline classifiers, given the 1444 features all measuring properties of the text, possible colinearity between features, and variance of features in the training data. We hypothesised that splitting the features into a number of subsets and deriving a range of principal components from these, rather than deriving principal components from all features at once, would reduce the number of false negative results by using a wider range of principal components. We therefore repeated the experiments from the baseline phase with a RF ensemble classifier.

Ensemble meta classifiers can incorporate a number of combined baseline classifiers. We experimented with incorporating all the classifiers used in the baseline experiments to determine how the principles of RF could improve these. As the initial results showed varying performance between classifiers - for example, the NB produced the lowest numbers of false positives using *Set*1 and *Set*3, but SVM produced the lowest false negatives in both cases - we chose to incorporate a second metaclassifier within the RF that used a voting principle as a mechanism to assign the label with maximum probability across all base classifiers to new instances. SVM, J48 Decision Tree and Naive Bayes classifiers were integrated within the RF classifier as an ensemble, with the classifier producing the maximum probability for new instances being selected for each classification decision. We ran two experiments with the RF approach – one with all three baseline classifiers and another with just NB and SVM classifiers. Table 3 shows the notable difference in performance when using DT to classify suicidal ideation, thus it was dropped and the ensemble approach performed much better. We have only reported the results of the NB and SVM combination.

**Table 3:** Machine classification results: suicidal ideation.

	Classifier				
Feature		NB	DT	SVM	RF
*Set*1	P	0.514	0.464	**0.657**	0.587
	R	*0.731*	0.410	0.564	0.474
	F	0.603	0.435	*0.607*	0.525
*Set*2	P	0.491	0.397	*0.652*	0.589
	R	*0.705*	0.372	0.577	0.423
	F	0.579	0.384	*0.612*	0.493
*Set*3	P	0.505	0.530	*0.647*	0.614
	R	*0.705*	0.449	0.564	0.449
	F	0.588	0.486	*0.603*	0.519
Combined	P	0.496	0.447	0.551	*0.644*
	R	0.718	0.487	0.692	**0.744**
	F	0.586	0.466	0.614	**0.690**
PCA	P	0.400	*0.446*	0.441	0.438
(combined)	R	0.590	0.526	0.385	*0.628*
	F	0.477	0.482	0.411	*0.516*

## 5 Results and evaluation

We used a 10-fold cross validation approach in the evaluation of our classification experiments. This approach iteratively trains the classifier on 90% of the training data and tests on the remaining 10%. After 10 iterations, the results are calculated by taking the mean accuracy across all models. The results are provided in this section at two levels. Tables 2 and dummyTXdummy- 3 present the results for each of the baseline classifiers – Naive Bayes (NB), J48 Decision Tree (DT), and Support Vector Machine (SVM). Each row represents the results using a different set of features. The final column in the table provides the results of the Rotation Forest (RF) ensemble classifier. Table 2 provides the weighted average results across all classes, while Table 3 provides the results of the key class of interest – suicidal ideation. Evaluation followed standard classification measures of *Precision* measuring false positives, *Recall* measuring false negatives, and *F-measure* a harmonized mean. In the Tables we represent the best scores in bold, and the best precision and recall for each feature set in italic.

The three baseline models perform similarly across all classes for feature set 1,2 and 3, with SVM slightly outperforming NB in most cases, and DT performing least well (see Table 2). In two out of 3 cases NB achieved the best precision score and SVM the best recall in all three – leading us to test an ensemble approach. It is interesting to note that combining all feature sets led to only a 0.001 improvement in precision and actually reduced recall by 0.07 when compared to *Set 2*. Furthermore, applying a dimension reduction method - Principle Component Analysis (PCA) – led to a further reduction in performance when applied to all features (see bottom three rows of Table 2 and dummyTXdummy- 3). This is likely because PCA reduces the number of features available to the models. However, when the training data was split into smaller samples, with principle components derived for each sample via the Rotation Forest method – thus broadening the diversity of components while retaining complexity – we saw a performance increase, going from a maximum performance of P = 0.695 and R = 0.689 to P = 0.732 and R = 0.729 across all classes when applying the RF approach combined with a Maximum Likelihood voting metaclassifier.

When digging deeper into the key class of interest – the suicidal ideation class – we see a reduced performance for all base classifiers (see Table 3). The confusion matrix for the best performing classification model (see Table 4) shows that this is largely due to confusion between c1 (suicidal ideation) and c3 (flippant reference to suicide). This was always going to be a challenge given the subjective nature of the task and the difficulty human annotators found in agreeing on this. Sarcasm and irony are notable text classification challenges that are yet to be resolved. This is primarily due to the same language often being used in serious and flippant cases. However, the SVM baseline classifier still achieved a Precision performance of 0.657, which was in fact the best performance - even better than the RF classifier. Indeed, the baseline SVM generally outperformed the other base classifiers, and the RF ensemble, for the individual sets of features. This is in line with other existing research in this area, though we have achieved a higher performance. Yet when combining all features, and applying principle component analysis to smaller subsets of training data, the RF model performed significantly better than any other classification model for the suicidal ideation class. The maximum Recall was 0.744, which is only a slight improvement of 0.013 over the NB baseline using *Set 1*, but the maximum F-measure was 0.69 as compared to 0.61. These results suggest that the ensemble of multiple base classifiers with a maximum probability meta classifier offers a promising way forward for the multi-class classification of suicidal communication and ideation in ’noisy’ short informal text, such as social media posts. The ’none of the above’ confusion also suggests there may be other latent topics not present in our set of class labels. Identifying these may be a useful task for future research. Table 5 provides P, R and F results for the best performing classifier across all classes for comparison.

**Table 4:** Confusion matrix for the best performing classification model.

Class	c1	c2	c3	c4	c5	c6	c7
c1	***58***	0	15	0	0	0	5
c2	0	***18***	1	4	0	4	1
c3	11	0	***143***	0	1	5	17
c4	0	4	5	***18***	0	2	6
c5	1	1	1	0	***31***	1	1
c6	0	6	9	7	2	***76***	4
c7	20	0	23	0	2	4	***94***

**Table 5:** Precision, recall, and F-measure for the best performing classification model.

class	P	R	F
c1	0.644	0.744	0.690
c2	0.621	0.643	0.631
c3	0.726	0.808	0.765
c4	0.621	0.514	0.563
c5	0.861	0.861	0.861
c6	0.826	0.731	0.776
c7	0.734	0.657	0.694

## 6 12 month case study of machine classification and real-world events

Once trained and tested we applied the best performing machine classifier to data collected from Twitter for a 12 month period from 1st February 2014 to 31st January 2015. A geographical filter was applied to restrict the tweets analysed to those likely to originate in England. Two methods were used for this. Firstly those accounts selecting the London time zone were included. This time zone includes all of the UK and the Republic of Ireland, but around four-fifths of the population of the British Isles live in England. It should be noted that there will also be some errors wherein Twitter users have selected the London time zone despite not living there. The second method was to match information in Twitter user profiles with a list of English counties. Those with US equivalents (e.g. Lincoln) had to be excluded. These were initially identified manually and then automated removal was used for US accounts with these locations. During this 12-month period there were several real-world events related to suicidal communication – most relevant being the death of actor Robin Williams, which was reported as suicide in global media.

One of the major concerns with machine classification is the generalizability of the learned model beyond the training and testing sample. To extend the classification experiments and test the utility of the classifier for a much larger sample we selected a systematic sample of tweets from the 12 months collection, repeated the human annotation tasks, and report the performance of the classifier on this much larger sample of previously unseen content. Furthermore, we plot the human annotated sample over the 12 month period to demonstrate the applicability of the classifier results for quantifying and visualizing public communication on suicide-related topics – particularly looking for spikes in communication from individual classes such as evidence of suicidal ideation.

### 6.1 Accuracy of the classifier

We split the 12-month study into two tasks – a binary classification (’is this person suicidal?’) and the 7-class task to classify text in accordance with a more nuanced and fine-grained framework. For the binary task we took a sample of 2000 tweets classified by our ensemble method and asked human annotators to label the outputs. Of the 2000 tweets the human annotators’ agreement on labels was above our threshold of 75% agreement in 1731 cases. 282 of these were labelled by our classifier as ’suicidal’. 240 of the 282 were agreed to be suicidal by the human annotators, giving an accuracy of 85%. That is, over a 12 month period, on a systematic sample of output from our classifier, 85% of the outputs of the automated task were confirmed to be suicidal in nature by at least 3 out of 4 human annotators.

For the 7-class task we took another systematic sample of 2000 tweets and produced outputs from the machine classifier according to the seven different classes. We invoked the human annotation tasks again and identified that only 1121 had agreement between 3 out of the 4 annotators, suggesting the task is increasingly complex over longer periods with multiple contributing events. 805 tweets were only agreed by two annotators, and 74 were not agreed at all. Table 6 provides a confusion matrix for the 12 months sample. For the suicidal ideation class (class 1) we can see a total of 170 tweets were assigned the label by human annotators, 111 of these were classified correctly by our ensemble method – an accuracy of 65.29%., which is within 1% of the original sample result derived from the confusion matrix in Table 4. This provides evidence of the consistency in the classifier results over a 12 month period within which different events, language and platform alterations have occurred.

**Table 6:** Confusion matrix for classification model applied over 12 months.

Class	c1	c2	c3	c4	c5	c6	c7
c1	***111***	0	15	3	0	39	2
c2	4	***19***	6	93	1	26	16
c3	22	0	***64***	14	1	30	10
c4	0	16	1	***140***	0	12	17
c5	11	0	21	27	***3***	43	45
c6	28	1	18	12	0	***96***	13
c7	2	3	7	35	1	28	***64***

### 6.2 The prevalence of different types of suicide-related communication

Over the 12 months when Twitter was monitored, 1,884,248 tweets were collected which contained the 62 keywords and were ostensibly from England or in the London time zone. The classifier was applied to this sample. The most common category of suicide-related content (48%) was flippant reference to suicide. This is when apparently serious statements such as ‘I want to kill myself’ are made in relation to something patently trivial such as a television programme the Twitter user dislikes or the failure to find a favourite brand of crisps. The next most common category was tweets which although containing one or more of the 62 keywords were classified as not in any of the categories of suicide-related communication (35.4%). All the five other categories were comparatively much less numerous (see Table 7).

**Table 7:** Classification of tweets from England and the London time zone over 12 months - initially filtered using suicide-related keywords.

Category of	N of i	%	Mean	Standard
suicide-related	tweets		daily	deviation
communication.	in 12m		rate	
Possible suicidal intent	108,195	5.7	296	122
Flippant reference to suicide	904,373	48.0	2478	2380
Information and support	59,204	3.1	162	223
Memorial	34,588	1.8	95	111
Campaigning	3,699	0.2	10	19
Reporting news of				
a suicide (not bombing)	106,741	5.7	292	697
None of the above	667,448	35.4	1829	935
Total	1,884,248	100		

### 6.3 Suicidal ideation in Twitter in England over a 12-month period

**Fig. 1:**
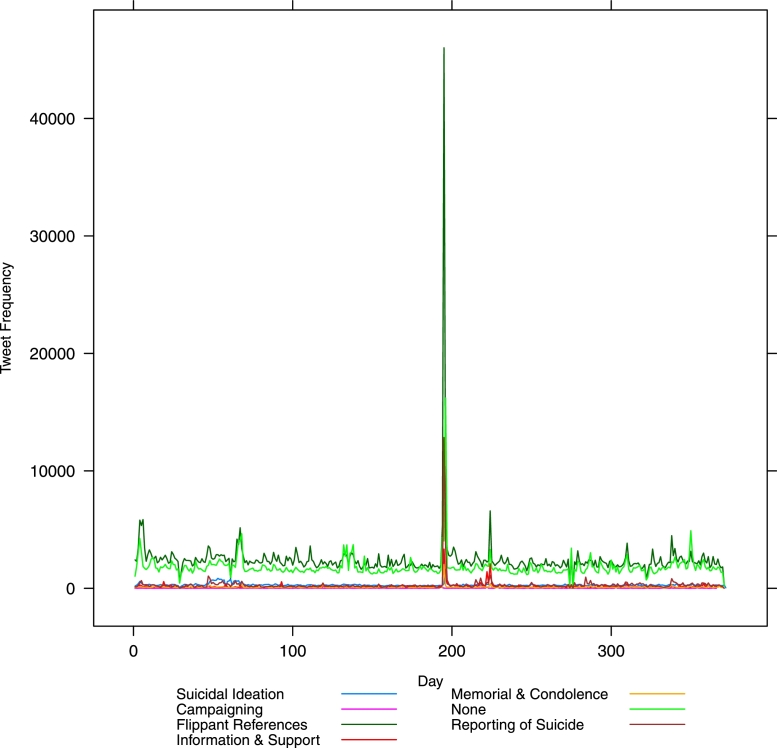
Class-wise frequency for all classes over time – in day units from 1*st* February 2014.

**Fig. 2:**
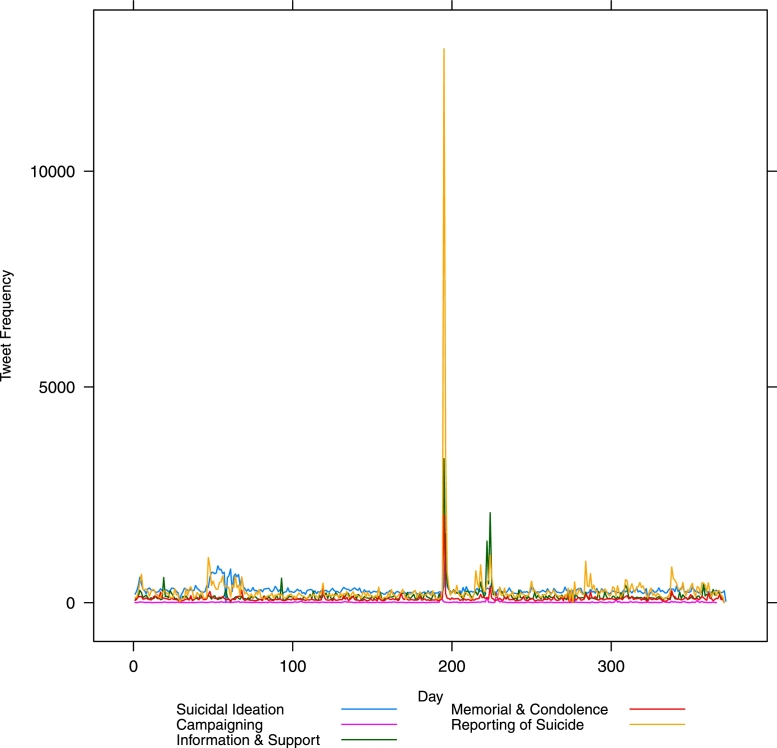
Class-wise frequency without flippancy or no relevance over time – in day units from 1*st* February 2014.

**Fig. 3:**
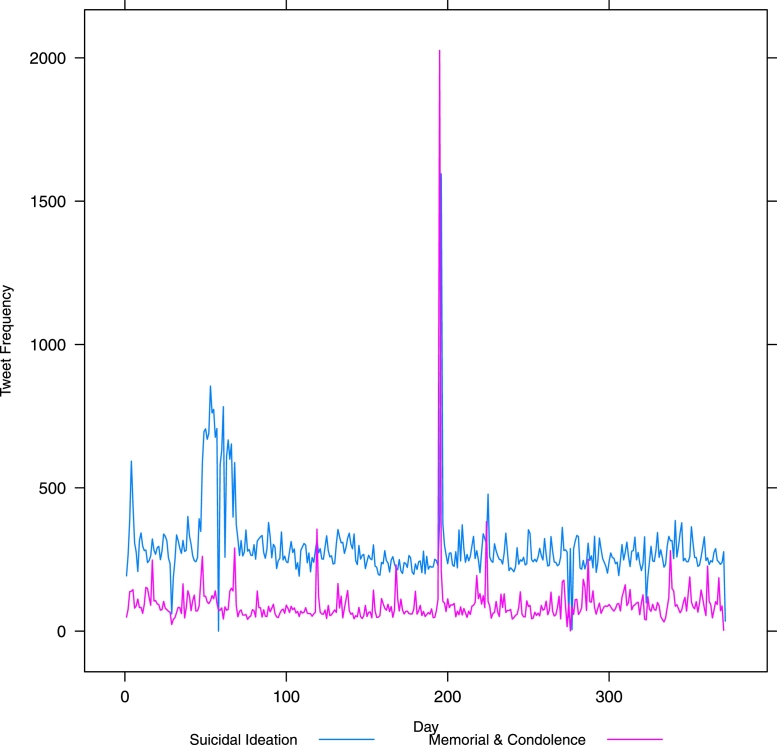
Class-wise frequency for suicidal ideation and memorial over time – in day units from 1*st* February 2014.

Fig. 1 presents a plot of the fluctuation of tweets over the 12 month period February 2014–January 2015 for all classes as labelled by our machine classifier. Fig. 2 shows the same but without the flippant references and non-relevant tweets. Fig. 3 drops out everything except suicidal ideation and memorial or condolence. It is likely that the visible peaks in Fig. 3 relate to widely publicized celebrity suicides.

The large peak (over 1500 tweets) around the time of the actor Robin Williams’s death on 11 August 2014 is very clear (see x =  ∼ 200 in Fig. 2). Less obvious is a peak around the time of actor Phillip Seymour Hoffman’s death on 2 February 2014 (x = ∼2−5 in Fig. 2). There is also an apparent peak following the death of fashion designer L’Wren Scott on 17 March 2014 (x = ∼45−50 in Fig. 2), lasting several days. Of note here is the utility of this method for policy makers and those responsible for public safety and well-being in times of heightened risk, such as time of widely publicized suicides. Note that these charts are automatically generated by the classifier, with no human resource required, and we have provided evidence as to the stability of the classification results on a systematic sample of data collected over a 12 month period.

In Fig. 2 there is a massive spike in reporting of suicides around day 200 (see x-axis) when the news of Robin Williams’ suicide broke. Suicidal ideation and memorials also spike at the same time (see x =  ∼ 200 in Fig. 3). This is to be expected. However, note the other spikes in Fig. 3 – especially the sustained spike early on in the year (x = ∼50−75). Unlike the spike following Williams’ death where a spike in information and support also occurred (see x = ∼50−75 in Fig. 2), the earlier spikes in suicidal ideation are (i) more extended over a longer period, and (ii) do not exhibit an equivalent spike in information and support. We posit that these are two aspects that potentially increase the risk to social media users and warrant further investigation by the relevant bodies.

### 6.4 The demographic characteristics of accounts tweeting suicide-related content: age and gender

The method developed in [54] to determine gender of Twitter user from profile information was applied to the tweets categorised by our classifier as indicating possible suicidal intent. This method involved matching a very large file of first names world-wide 40,000 *namen* (German for ‘names’) [55] to information in Twitter users’ profiles. The names are categorised as male, female or unisex. Only the first name in the Twitter profile was used, on the assumption that most UK users would put their first name first. If the first name was a substring of one or more of the names in the names file, the identity of the users was determined on the basis of the gender with the greatest count.

Out of a total of 43185 users, 22742 (52.7%) contained names which matched entries in the names database. A further 20443 (47.3%) could not be matched. Of those which could, 6846 (30.1%) were male, 10394 (45.7%) were female and 5502 (24.2%) were unisex. This compares with the proportions found in a random sample of 13 million Twitter accounts of 45% male, 47% female and 8% unisex [54]. There is apparently an under-representation of men using the language of suicidal intent in Twitter and an over-representation of users with unisex names.

The method proposed in [56] for determining age in Twitter was also applied. This is a pattern-matching approach using variants of the following phrases for age extraction: 1. I am X years old 2. Born in X 3. X years old with X being either a two-digit number or a date in various forms from which age is calculated. Additional terms were used to remove false positives, as described by [56].

It is interesting to note that of those users tweeting possible suicidal intent whose ages could be detected using this method, 38 (10.5%) were identified as being under 13. However, a better comparison can be made with general Twitter use by comparing only the users aged 13+, as this is the lower age bar used in the baseline study in [56] on the grounds that to access a Twitter account users are supposed to be at least aged 13. Table 8 shows the comparative percentages of users aged 13+ whose tweets indicated possible suicidal intent. The teenage category is over-represented, compared with the baseline study of Twitter users.

**Table 8:** Age group of Twitter users tweeting possible suicidal intent (age 13+) and comparison with all Twitter users.

Age	Number	%	%
group	of apparently suicidal tweets	share	share in all Twitter
13–20	345	81.2	59.4
21–30	71	16.7	31.6
31–40	7	1.6	4.4
41–50	2	0.5	3.4
51–60	0	0	1.1
60+	0	0	0.3
Total	425	100	

This method of course only works with users who state their age and it is very important to note that older age groups are much less likely to state their age or use Twitter. Nonetheless, the same limitations apply to the current study and the larger study of all Twitter users proposed in [56] so a comparison with the findings of that study is still illuminating.

#### 6.4.1 Correlation between tweet rate and suicide rate

The Office for National Statistics (ONS) provided daily counts of deaths, by age and sex, in England and Wales which coroners had determined as suicides and deaths by injury/poisoning of undetermined intent for the 12-month period during which Twitter was monitored (1.2.14 – 31.1.15). Both types of death combined are referred to here as ‘suicides’. Spearman’s rank correlation coefficients were calculated to test for any relationship between the daily suicide rate and the rate of suicidal tweets, both for the day of death (24 hours) and also for the 48 hours following the death. Tests were conducted for all deaths and also for just female deaths and under-35 deaths, given what was suggested above about the demographic patterns of suicidal tweeting. For a 24-hour period, the correlations were as follows: for all deaths r = 0.06 (p = 0.26), for under-35s r = -0.00 (p = 0.97) and for females r = 0.11 (p = 0.03). For a 48-hour period the correlations were these: for all deaths r = 0.06 (p = 0.21), for under-35 s r = 0.03 (p = 0.57) and for females r = 0.06 (p = 0.24). There was therefore some evidence against the null hypothesis that the tweet rate in the 24 hours in which the suicide took place was independent of the daily female suicide rate. However, this correlation was very weak and there was no evidence of any correlation for any other category of death.

## 7 Discussion

In this section we analyse the components produced by running the Principle Component Analysis (PCA) method on the *combined* set that resulted in the best set of results, as shown in Tables 2–5. We also reflect on the application of the classifier to Twitter data collected over 12 months.

The application of PCA reduced the features set from 1444 to 255 attributes in terms of main components. For the seven suicide-related classes we show in Tables 9 and 10 the most representative principal components and briefly discuss what each class represents in terms of the features in the component and the particular language used in it.

**Table 9:** Principal components per class.

**Table 10:** Principal components per class.

Note that while the distribution of the components per class mirrors the total number of annotation per class (therefore penalising the classes less represented in our data set such as ‘memorials’) in Tables 9 and 10 and in the related discussion we are giving priority to the most representative class of posts containing evidence of possible suicidal intent. We can observe the following characteristics of the features included for each class component:

*c1:* Many of the features that appear dominant in the suicidal ideation class are those related to phrases and expressions identified in the suicide literature as being significantly associated within the language of suicide. In particular, beside a limited number of uni/bi/tri-grams generated directly from the training set, the terms derived from a number of suicide related Web sites were fundamental in classifying suicidal ideation in Twitter data. As were the regular expression features derived from Tumblr posts. Examples like ‘end it all now’ and ‘want to be dead’ and regex including expression of ‘depressive/suicidal/self harming’ ...‘thoughts /feelings’ appear strongly related to suicidal ideation and are clearly discriminating for this specific class. Other terms (such as ‘killing myself’ and the regex containing ‘die’ ... ‘my sleep’) become effective for classification when used besides other attributes such as lexical features that express surprise, exaggeration and emphasis (e.g. adverbs (‘really’), predeterminers (e.g. ‘such’ ‘rather’)), and words mapped to specific ‘affective’ domains such as ‘alarm’ and ‘misery’. Note that some other concepts and terms appear with a negative correlation as expressions of opposite affective states, such as ‘security’ and ‘admiration’.

*c2:* For the class representing campaigning and petitions we can observe more general concepts, again expressed by regular expressions and language clues (word-lists in our terminology), such as ‘support/help’, ‘blog’ as well as more specific terms (e.g ‘safety plea’) and expressions (‘put an end to this’). Some of the Wordnet domain features require further examination as they appear confusing at first – for example ‘racing’ is picking up on the words ‘run’ and ‘running’ that are related to campaigns.

*c3:* As the confusion matrix in Table 4 shows, the class concerning a ‘flippant’ use of suicidal language is the one presenting the major difficulties in classification, since it includes many of the same linguistic features of suicidal ideation. However, the principal components derived for this class identify certain attributes that are the opposite type of sentiment from emotional distress. These include affective states such as ‘levity’, ‘gaiety’, ‘jollity’, and ‘cheerfulness’, as well as popular conversational topics, such as casual remarks about the weather. The confusion occurs where phrases such as ‘kill myself’ are used frivolously.

*c4:* The class representing posts related to information and support (and prevention) appear mostly represented by specific words (often unigrams and ‘tags’) directly linked to the support services (e.g. #police, #officers, internet and suicide) and/or topicality (such as sexual references (‘#lgtb), and the domains of self-harm and #suicide).

*c5:* For the class concerning memorial messages, as may be expected, direct mentions of the name of the deceased appear highly influential as well as ‘time’ references (e.g. ‘a month ago’, ‘a year since’) in association with terms such as ‘killed’ and ’died’ (well captured by one of our regular expressions). In addition labels and tags as ‘rip’ and terms expressing ‘love’ ‘and ‘affection’ are also part of the components associated with this class. Again, we see some Wordnet domains appearing - ‘mathematics’ and ‘agriculture’ are related to specific words such as ‘add’ and ‘grow’.

*c6:* The class concerning news reports related to suicide presents features such as words representing sources of information (e.g. #bbc news), types of news (research study or statistical report), and direct mentions of the name of the deceased (as well as general concepts related to the particular case, such as in the one here reported of the ‘TV’ domain). Note that the last three classes of memorial, information/support, and news reporting all share the common characteristics of including URL links within the tweets which, consequently, does not result in an effective feature for discrimination between these different classes.

*c7:* Finally, the class of posts annotated as not related to any of the previous classes exhibits attributes such as general phrases related to self doubt (such as ‘what’s wrong with me and ‘hate myself’) and emotional states (such as ‘jitteriness’ and ‘admiration’). These are phrases that could appear in tweets relating to emotional distress but are also clearly evident in general everyday ‘chatter’.

The monitoring of Twitter over 12 months found that the frequency of apparently suicidal statements seemed to increase around the time of high-profile celebrity suicides. Although we cannot claim from our data that suicidal statements online map directly onto offline behaviour, previous studies have shown an association between celebrity suicide reporting and actual suicide rates (see the meta-analysis in [36]. Whilst acknowledging the limitations of the demographic analysis, especially for age, where only the youngest twitter users are likely to give any indication of how old they are, it is interesting to note that there seem to be more women than men tweeting apparently suicidal statement and when compared with studies of all twitter usage (using the same method), there may be a comparatively younger age group of twitter users tweeting these statements, especially teenagers. The gender profile fits with the broader picture of the gendered communication of suicidality [57] with women more likely to attempt suicide and express suicidal ideation than men, despite the higher rate of fatality in men – what has been termed the ‘gender paradox’ of suicidal behaviour [58]. The general lack of correlation between the rate of suicidal tweeting and the daily suicide rate in England might suggest the online expression of suicidal thoughts is a distinct phenomenon that is unconnected to offline behaviour. However the rate of suicides per day is likely to be too narrow a time-frame and future studies should collect social media and suicide data over a longer period to allow for consideration of the correlation of online and offline suicidality using a monthly rate. It should be noted that even if suicidal statements in social media are not necessarily an indicator of immediate risk of suicide, they very likely do suggest these individuals are distressed and in need of support. Also, the online expression of suicidal feelings may well suggest longer-term risk of suicide.

## 8 Conclusion

In this paper we developed a number of machine classification models built with the aim of classifying text relating to communications around suicide on Twitter. The classifier distinguishes between the more worrying content, such as suicidal ideation, and other suicide-related topics such as reporting of a suicide, memorial, campaigning and support. We built a set of baseline classifiers using lexical, structural, emotive and psychological features extracted from Twitter posts. We then improved on the baseline classifiers by building an ensemble classifier using the Rotation Forest algorithm, achieving an F-measure of 0.728 overall (for 7 classes, including suicidal ideation) and 0.69 for the suicidal ideation class.

We summarised and attempted to explain the results by reflecting on the most significant predictive principle components of each class to provide insight into the language used on Twitter around suicide-related communication. From this analysis we observed that *word-lists and regular expressions (regex)* extracted from online suicide-related discussion fora and other microblogging Web sites appear capable of capturing relevant language ‘clues’, both in terms of single words, n-grams (word-lists) and more complex patterns.These appear particularly effective for the suicidal ideation class, expressing emotional distress. *Lexical and grammar features* such as POSs appear mostly ineffective and scarcely present in the principal components (only some mentions as predeterminers, existential clauses and superlatives that, however, also relate to more specific ‘affective’ language features than only pure lexical ones). *Affective lexical domains*, appear instead very relevant (such as those represented by the WordNet library of ‘cognitive synonyms’) and able to well represent the affective and emotional states associated to this particular type of language.

Concepts and labels representing broader semantic domains (also derived form the WordNet library) are, on the contrary, not effective. In fact, although they appear rather numerous as attributes within the principle components they reveal to be, on close inspection, for the majority of cases irrelevant and mostly generated by a ‘confusion’ and ‘mis-representation’ of words (such as sentences like ‘my reason crashed’ associated to the ‘motor-racing’ domain, and ‘suicide watch’ associated to ‘numismatic’).

*Sentiment Scores* generated by software tools for sentiment analysis appear also ineffective and either scarcely or not at all included within the principal features of each class. Note that this is true for both basic tools that only provide a binary representation of positive and negative score values (SentiWordNet) as well as more sophisticated text analysis software that generate sentiment scores over a larger range of labels representing emotional states (LIWC).

A classifier for suicide-related language could potentially make an important contribution to suicide prevention. Monitoring individual social media accounts via keywords that suggest possible suicidal ideation is controversial territory, as shown by the recent withdrawal of the Samaritans Radar app in the UK^11^ but there is nonetheless potential for such a lexicon to contribute to prevention in some way, as long as acceptability to social media users is thoroughly investigated. The ‘real-time’ identification of aggregate levels of suicide-related communication at scale in online social networks, which could be facilitated by the ensemble classifier produced in this research, is one possible approach. There is positive potential, for example, for using the classifier to monitor trends at an aggregate level, to inform service provision. Although we found a lack of correlation between the timing of apparently suicidal tweets and actual suicides, nonetheless, a marked increase in the volume of suicidal tweets, such as around the time of high profile celebrity suicides, may well suggest an increased need for helpline and other support for people who are in distress and perhaps at longer-term risk of suicide. Using the classifier to monitor social media communication could help with planning for increased provision.

Our classifier goes beyond the recognition of suicidal language insofar as it also aids identification of other kinds of communication, in recognition that social media platforms can be used for multiple purposes, including the reporting of news and marshalling of campaigns. Monitoring of suicide news reporting in social media is another potential avenue where text mining and machine classification techniques could be applied. The identification of flippant use of suicidal language could be especially useful. The methods needs further development, ideally with a larger sample of social media postings, and application to platforms other than Twitter. Finally, we note that it is important to retain collaboration with domain experts in suicidology throughout the experimental and interpretation phases of future research to improve classification accuracy by incorporating prior knowledge of the characteristics of suicidal language - especially given the significance of the affective features in this paper.

## Footnotes

1 http://www.experienceproject.com.

2 http://www.enotalone.com.

3 http://www.takethislife.com.

4 http://www.recoveryourlife.com.

5 https://www.tumblr.com.

6 http://www.crowdflower.com.

7 http://nlp.stanford.edu/software/tagger.shtml.

8 http://wndomains.fbk.eu.

9 http://sentiwordnet.isti.cnr.it.

10 http://www.cs.waikato.ac.nz/ml/weka/.

11 http://www.bbc.co.uk/news/technology-29962199.
